# Ultrasonography of fibroma of the tendon sheath in the hand and wrist

**DOI:** 10.1186/s12891-023-06250-y

**Published:** 2023-02-23

**Authors:** Yibing Zhao, Yi Ding, Tao Chen

**Affiliations:** 1 Department of Ultrasound, Beijing Jishuitan Hospital, Fourth Clinical College of Peking University, No.31 Xinjiekou Dongjie, Xicheng District, Beijing, 100035 China; 2 Department of Pathology, Beijing Jishuitan Hospital, Fourth Clinical College of Peking University, Beijing, China

**Keywords:** Fibroma of the tendon sheath, Sonography, Hand and wrist

## Abstract

**Background:**

The purpose of our study was to explore the sonographic characteristics of fibromas of the tendon sheath of the hand and wrist and to evaluate the value of high frequency ultrasound in the diagnosis of FTS.

**Methods:**

We retrospectively reviewed the sonography of 42 patients with surgically proven FTS, including one with a relapsing tumor (43 lesions in total). The location, size, distribution, relationship with the surrounding tissue, two-dimensional gray-scale sonographic appearance and internal color blood flow of all lesions were analysed.

**Results:**

The maximum diameter ranged from 0.4 to 2.8 cm, with an average of 1.5 ± 0.6 cm. Twenty-eight lesions (65%) were associated with an adjacent tendon, while the other 15 lesions (35%) were next to the joint. Spindle or oval lesions were common, followed by irregular shape. The nodules with clear boundaries were hypoechoic and had posterior echo enhancement. Thirty-seven lesions (86%) were homogeneous, while 6 lesions (14%) had cystic components with no echo inside. Seventeen lesions (40%) had a large amount of blood flow. Nine lesions (20%) had a small amount of blood flow. The other 17 lesions (40%) had no significant blood flow.

**Conclusions:**

The diagnosis of fibroma of the tendon sheath can be considered when ultrasound examination reveals a focal nodular mass adjacent to a tendon sheath with homogeneous hypoechogenicity and no or small or large amounts of blood flow.

## Background

Fibroma of the tendon sheath (FTS) is a rare, benign tumor that is firmly attached to the tendon sheath and is composed of tightly packed spindle cells surrounded by collagen fibers. It occurs more commonly in the extremities, particularly in the hands and wrists. Chung and Enzinger published a report of approximately 138 cases of FTS in 1979 [1], which was the largest series on FTS and the basis of our understanding of this disease. Fox MG et al. studied MR imaging of fibromas of the tendon sheath [2, 3]. However, there is little literature on the diagnosis of FTS by ultrasound [4–7]. This study analysed FTS based on 43 lesions confirmed by surgery and pathology, with the aim of exploring the diagnostic value of ultrasonography in fibroma of the tendon sheath.

## Methods

### Participants

A retrospective analysis involving ultrasonography of FTS was performed from January 2015 to December 2021 in Beijing Jishuitan Hospital. This study included 42 patients who were confirmed to have FTS by surgery and pathology, there were 25 males and 17 females, they were aged 7 ~ 66 years, and the mean age was 36 ± 15 years.

### Imaging protocols and image analysis

All gray scale ultrasonographic examinations were conducted by a musculoskeletal radiologist with 10 years of experience using the Phillips IU22, EPIQ5, and EPIQ7 ultrasound instruments and an 18–20 MHz broadband linear array transducer. The shear wave elastography (SWE) ultrasonographic examinations were performed on the Airplorer ultrasound system and a 15 MHz linear array transducer. The location, size, distribution, relationship with the surrounding tissue, two-dimensional gray scale and internal color flow of all the lesions were analysed. The level of vascularization was classified according to Adler’s grades of blood flow signals: class 0 was no blood flow signals in the tumors; class I was 1–2 dot-like or fine rod-like blood flow signals in the tumors; class II was 3–4 dot-like or one important blood vessel, whose length was close to or exceeded the radius of the tumors; and class III was more than 5 dot-like or 2 longer blood vessels.

## Results

In this group of 42 patients, 41 patients had a solitary nodule and one patient had multiple (43 lesions in total). Tumor size ranged from 0.4 to 2.8 cm, with a mean of 1.4 ± 0.7 cm. Twenty-five patients (61%) had local painless slow-growing tumor. Sixteen patients (39%) had tenderness, of whom two (5%) had radiating pain. Two cases (5%) had a history of local trauma.

**Fig. 1:**
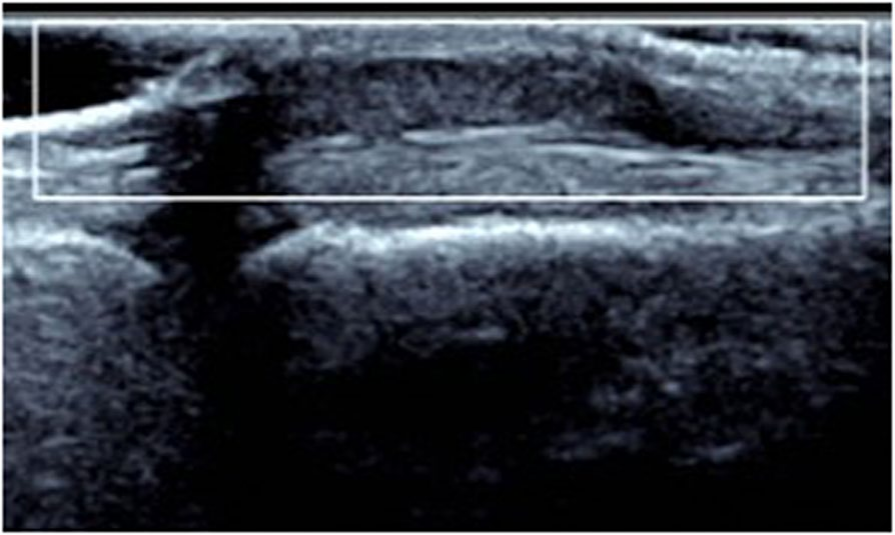
A 29-year-old man with a painless mass on the flexor surface of the thumb. Sonography shows a homogeneous hypoechoic nodule with no blood flow (Class 0)

**Fig. 2:**
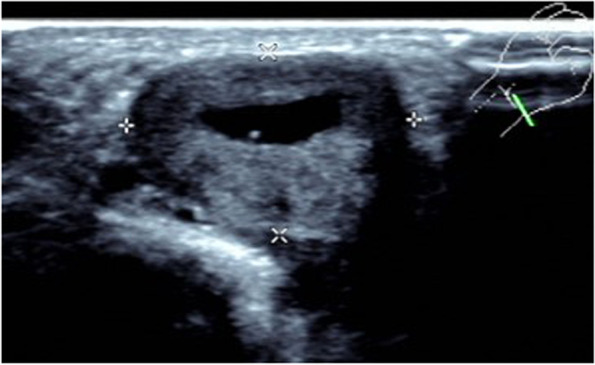
A 28-year-old man with a painless mass on the extensor surface of the wrist. Sonography shows an inhomogeneous hypoechoic nodule with an anechoic cystic area inside

**Fig. 3:**
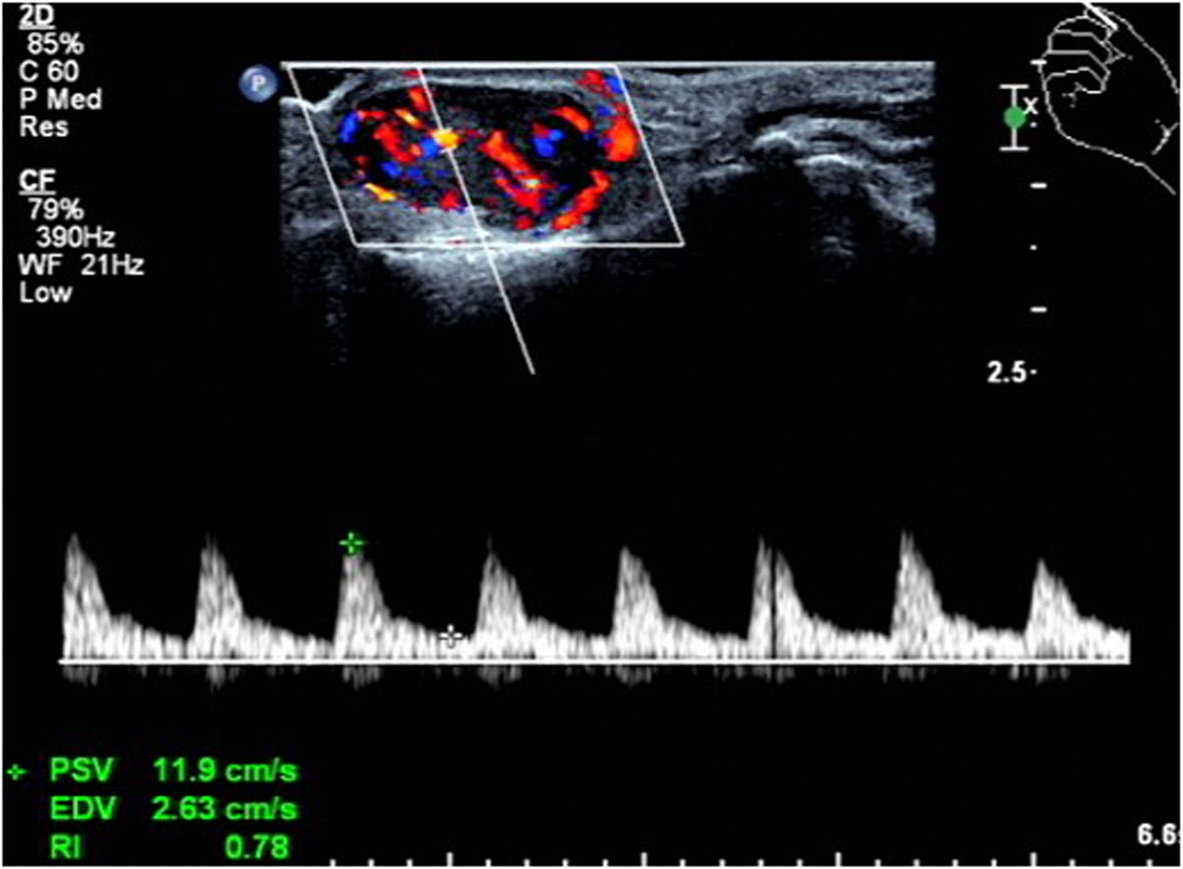
A 67-year-old man with a painless mass on the radialis surface of the wrist. Sonography shows a homogeneous hypoechoic nodule with abundant blood flow (Class III) and the arterial flow spectrum is detected

**Fig. 4:**
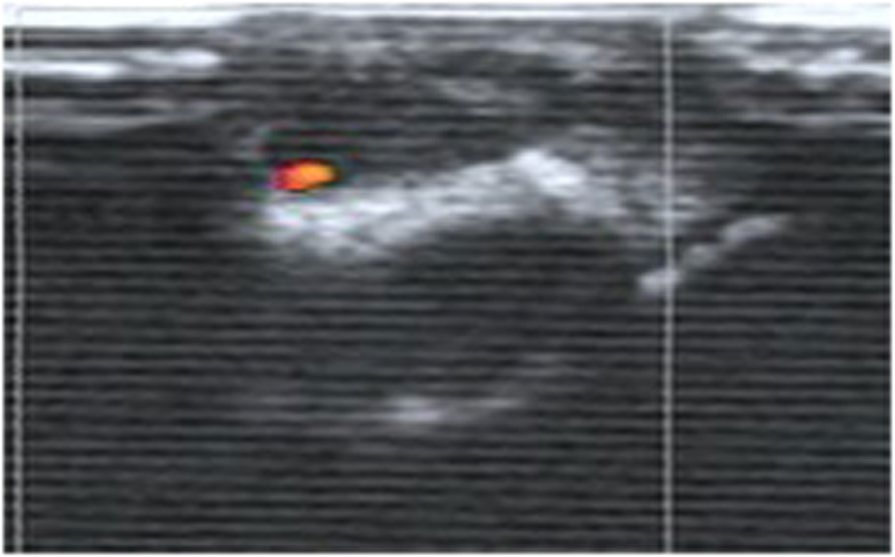
A 24-year-old man with a painless mass on the flexor surface of the index finger. Sonography shows a homogeneous hypoechoic nodule with a small amount of blood flow (Class I)

The sonographic manifestations of the patients in this group had certain characteristics: 28 lesions (65%) were associated with the adjacent tendon, of which 22 lesions (51%) were in the proximal fingers and 6 lesions (14%) were in the distal fingers. Of these, 20 lesions (47%) were associated with the flexor tendons of the fingers, while 8 lesions (18%) were next to the extensor tendon of the fingers. The other 15 lesions (35%) were next to the joint, of which 10 lesions (23%) were in the wrist and 5 lesions (12%) were next to the metacarpophalangeal joint and interphalangeal joint. Spindle or oval lesions were common, followed by irregular shape. The size of the hypoechoic nodules was no more than 3 cm, and they had clear boundary and posterior echo enhancement. Thirty-seven lesions (86%) were homogeneous (Fig. 1), while 6 (14%) lesions had cystic components with no echo inside **(**Fig. 2**)**. Seventeen lesions (40%) were class III (a large amount of blood flow)), of which 6 lesions (14%) could be detected in the arterial flow spectrum (Fig. 3). Nine lesions (20%) were class I (a small amount of blood flow) (Fig. 4), while the other 17 lesions (40%) were class 0 (no significant blood flow) (Fig. 1, Table 1).

**Table 1:** Location, relationship with the surrounding tissue and Adler grade of blood flow in 16 patients with fibroma of the tendon sheath

Location		Relationship with the surrounding tissue	Adler grades of blood flow	Number of case
Thumb	Proximal flexion	tendon	Grade III, with arterial flow spectrum	2
	Proximal flexion	tendon	Grade III	4
	Proximal radial	metacarpophalangeal joint	Grade III	2
	Proximal flexion	tendon	Grade 0	1
Index finger	Proximal flexion	tendon	Grade III, with arterial flow spectrum	2
	Proximal extension	tendon	Grade 0	2
	Proximal flexion	tendon	Grade I	2
Middle finger	Proximal radial	metacarpophalangeal joint	Grade I	2
	Proximal extension	tendon	Grade 0	2
	Distal Flexion	tendon	Grade 0	3
Ring finger	Distal radial	interphalangeal joint	Grade 0	1
	Proximal extensor	tendon	Grade I	2
	Proximal flexion	tendon	Grade III	2
Little finger	Proximal Flexion	tendon	Grade 0	2
	Distal Flexion	tendon	Grade 0	2
	Proximal extensor	tendon	Grade 0	2
Wrist joint	extension	wrist	Grade III	3
	flexion	wrist	Grade III with arterial flow spectrum	2
	extension	wrist	Grade I	3
	extension	wrist	Grade 0	2

Gross pathology revealed lobulated tumor with a well-circumscribed, smooth surface, spindle or oval shape and a thin capsule or no capsule. The microscopic sections showed cellular lobules separated by cleft-like vascular spaces, variable cellularity with occasional multinuclear cells in the more cellular regions, and a dense collagenous stroma. Typical lesions had fewer cellular components within the lobules. There was a significant increase in the amount of collagen fibers in a few of the lesions. Some of the local lesions had interstitial oedema, myxoid degeneration and cystic degeneration.

## Discussion

Fibroma of the tendon sheath, also called synovial tendon sheath fibroma [8], consists of a benign proliferation of fibroblasts that are firmly attached to the tendon or tendon sheath and are sometimes attached to the articular capsule [9]. In the study that Chung and Enzinger published about 138 cases of FTS, and the peak age was in the third to fifth decades of life with a male preponderance. 98% of the FTS lesions occurred in the extremities, 82% of the lesions occurred in the upper extremities, particularly involved the fingers, the hand and the wrist, and mostly located on the flexor surfaces [1]. In the above respects, our study corresponded to the published demographic data, and we found that this tumor can also occur in children [10]. Patients often present with a painless mass, and they occasionally with tenderness. A few patients have a history of trauma. In our series, all the lesions were located in the upper limbs, and 39% of them presented with various degrees of tenderness. This ratio was slightly higher than that reported in the literature. The possible pathological explanation for this phenomenon was that the small neural plexuses invaginated into the surface of the lesions.

On ultrasound, thirty-seven lesions in our study showed a homogeneous hypoechoic nodule that was less than that of muscle, as would be exhibited in a mainly fibrous lesion. Six of these lesions also showed an inhomogeneous hypoechoic nodule with an anechoic area inside. Microscopic examinations of the six lesions showed predominantly cystic changes in the anechoic area. The imaging and microscopic features of the six cases have previously been reported in the literature [11]. In addition, some case reports reported that plain radiographs and sonographic examinations demonstrated that some masses contained punctate calcifications, and on pathology, the lesion showed some foci of chondroid and osseous metaplasia, which explained for the calcifications [4–6]. However, in our study, we did not find this phenomenon.

Fibromas of the tendon sheath had different extents of blood flow. In our study, 17 lesions showed no blood flow. The microscopic features of these lesions included increased cellularity, decreased vascular slit-like channels, myxoid change and fibrous tissue with areas of hyalinization [12]. The findings may account for the lack of blood flow. Nine lesions showed a spot of blood flow inside; 17 lesions showed abundant blood flow both internally and peripherally, as previously reported; and in 6 lesions the arterial flow spectrum could be detected. These sonographic appearances correlated microscopically to the richness of vascular slit-like channels within hyalinized stroma. The more blood vessel there are, the richer blood flow [10]. The findings likely account for the different degrees of blood flow. Some reports also revealed a similar situation to that of our cases, in which blood flow varied from none to little to much [3, 4, 6].

**Fig. 5:**
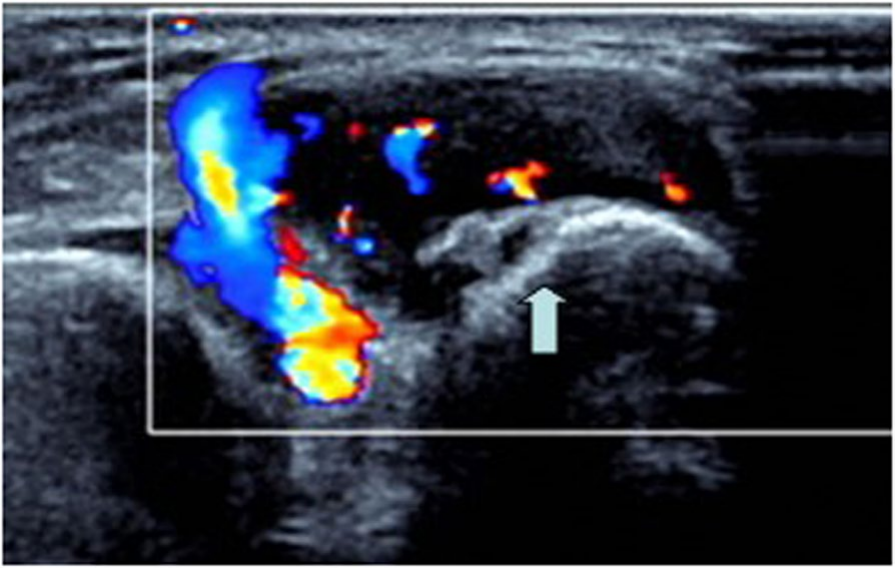
Axial sonogram of the index finger shows mild scalloping (arrow) of the flexor surface of the proximal phalanx

In addition to the above features, infrequent scalloping of the adjacent cortex due to pressure erosion from the tumor has been reported [13–15]. Mile erosion of the bone from the tumor appeared in one patient in our series (Fig. 5). In that patient, the lesion depressed the adjacent cortical bone. This finding is similar to that reported by Fox MG et al.

**Fig. 6:**
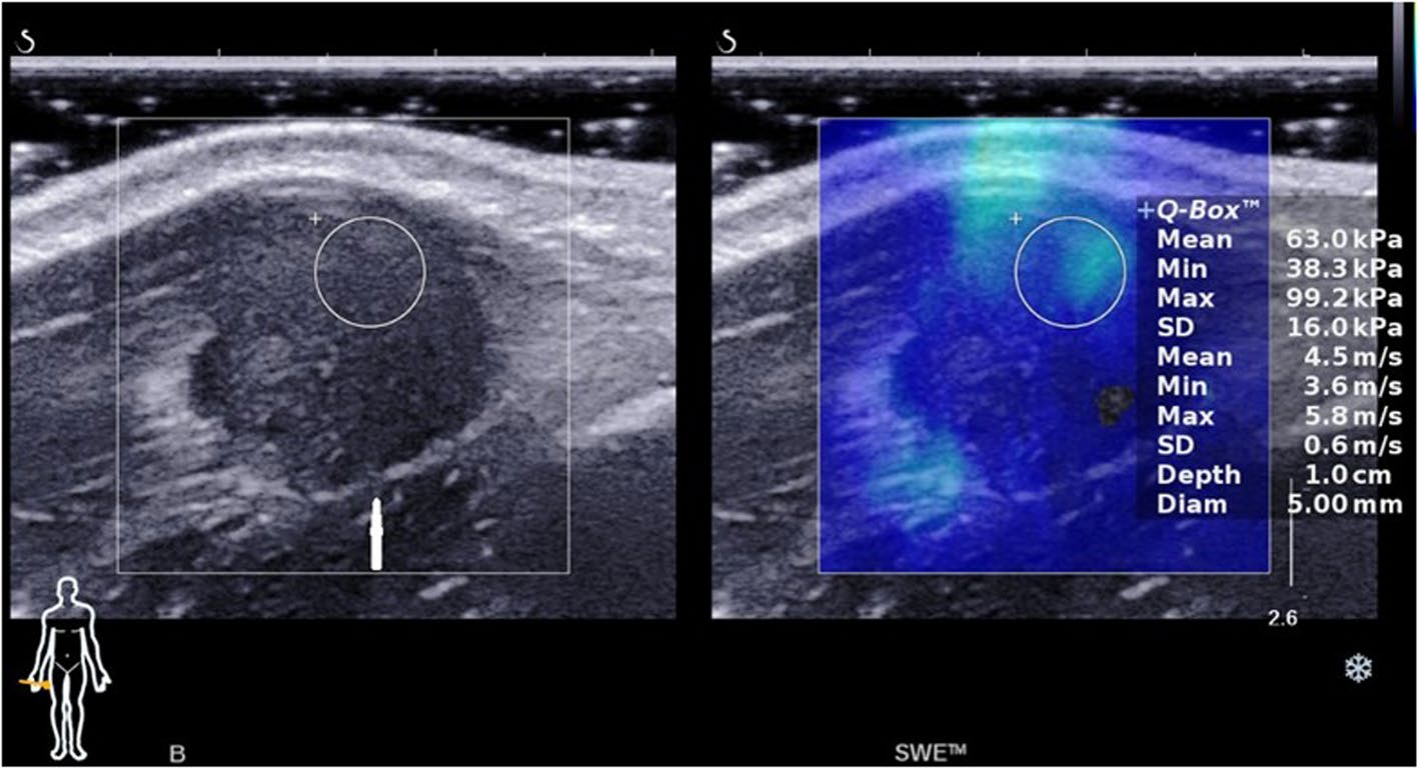
Gray-scale ultrasonography (left) shows a well-circumscribed homogeneous hypoechoic mass (arrows). Shear wave elastography (right) shows that the mean value of Young’s modulus is 63 kPa

There are no previous literature reports of the shear wave elastography findings of FTS. Ultrasound elastography techniques can provide valuable information about intrinsic properties by evaluating the tissue elasticity of soft tissue tumors [16]. According to our experimental data, the SWE findings of FTS indicated a hard tumor with a high value of shear modulus (E_mean_ is about 50 ~ 80 kPa) (Fig. 6), while E_mean_ of benign adipose tissue tumors is about 10 ~ 20 kPa, that of vascular tumors is about 15 ~ 30 kPa, and that of peripheral nerve tumors is about 25 ~ 45 kPa. It can be differentiated from benign adipose tissue tumors, vascular tumors and peripheral nerve tumors. The SWE findings can be considered highly useful for musculoskeletal diagnosis.

FTS is clinically rare, and it is easily misdiagnosed as other soft tissue tumors that are hypoechoic and occurs in the hands and wrist, for instance, giant cell tumor of the tendon sheath (GCTTS), nodular fasciitis, palm fibromatosis, schwannoma and so on [3, 17–19]. Among them, FTS is most often confused with giant cell tumor of the tendon sheath (GCTTS) at clinical examinations and on imaging [3, 20]. Both lesions occur in similar locations of the upper limbs, although GCTTS occurs more often than FTS [21]. Except that the shape of FTS is more regular, the ultrasonic findings of FTS and GCTTS are similar. So it must depend on pathology to confirm the final diagnosis.

In our study, the biggest limitation was the small size of the study group, which is the inherent limitation in a retrospective study. We look forwards to a follow-up study contains more data.

## Conclusions

In summary, a diagnosis of fibroma of the tendon sheath can be suggested when a painless slow-growing tumor occurs in a young to middle-aged individual, when a tumor is firmly located in the hand or wrist and is adjacent to the tendon or tendon sheath, and when sonography shows a focal nodular mass with homogeneous hypoechogenicity and no or little or much blood flow. Ultrasound examination has gained more popularity as a helpful technique for diagnosing FTS.

## Data Availability

Anonymized data from the present study will be shared on reasonable request from any qualified researcher for well-defined research questions. Please contact the corresponding author.

## Abbreviations

FTS: Fibroma of the tendon sheath
MR: Magnetic resonance
MHz: Megahertz
SWE: Shear wave elastography
GCTTS: Giant cell tumor of the tendon sheath

## References

[1] Chung EB, Enzinger FM. Fibroma of tendon sheath. Cancer. 1979;44(5):1945–54. 10.1002/1097-0142(197911)44:5<1945::aid-cncr2820440558>3.0.co;2-t. 10.1002/1097-0142(197911)44:5<1945::aid-cncr2820440558>3.0.co;2-t 91424

[2] Fox MG, Kransdorf MJ, Bancroft LW, Peterson JJ, Flemming DJ. MR imaging of fibroma of the tendon sheath. AJR Am J Roentgenol 2003;180(5):1449–145310.2214/ajr.180.5.1801449. 10.2214/ajr.180.5.1801449 12704067

[3] Ge Y, Guo G, You Y, Li Y, Xuan Y, Jin ZW, Yan G (2019). Magnetic resonance imaging features of fibromas and giant cell tum ors of the tendon sheath: differential diagnosis. Eur Radiol.

[4] Le Corroller T, Bouvier-Labit C, Sbihi A, Champsaur P (2008). Mineralized fibroma of the tendon sheath presenting as a bursitis. Skelet Radiol.

[5] Takakubo Y, Fukushima S, Asano T, Yamakawa M (2005). Case reports: intraarticular fibroma of the tendon sheath in the knee. Clin Orthop Relat Res.

[6] Hitora T, Yamamoto T, Akisue T, Marui T, Nagira K, Ohta R (2002). Fibroma of tendon sheath originating from the knee joint capsule. Clin Imaging.

[7] Jordan MM, Accomazzo R, Gaweda G, Zeri RS, Reisler T (2019). Fibroma of the tendon sheath arising from the flexor digitorum superficialis tendon. Eplasty..

[8] Shibayama H, Matsui Y, Kawamura D, Urita A, Ishii C, Kamishima T, Nishida M, Shimizu A, Iwasaki N (2020). Fibroma of tendon sheath of the hand in a 3-year-old boy: a case report. BMC Musculoskelet Disord.

[9] Plotkin B, Sampath SC, Sampath SC, Motamedi K (2016). MR imaging and US of the wrist tendons. Radiographics..

[10] Hermann G, Hoch BL, Springfield D, Abdelwahab IF, Klein MJ (2006). Intra-articular fibroma of tendon sheath of the shoulder joint: synovial fibroma. Skelet Radiol.

[11] Lu H, Chen Q, Shen H, Shen XQ, Wu SC (2016). Fibroma of tendon sheath in planta. Springerplus..

[12] Pulitzer DR, Martin PC, Reed RJ, Fibroma of tendon sheath (1989). A clinico-pathologic study of 32 cases. Am J Surg Pathol.

[13] Okada J, Shinozaki T, Hirato J, Yanagawa T, Takagishi K (2009). Fibroma of tendon sheath of the infrapatellar fat pad in the knee. Clin Imaging.

[14] Ahn JH, Lee YS, Lee DH, Ha HC (2008). Intraarticular fibroma of the posterior compartment in the knee: a case report. Knee..

[15] Moretti VM, de la Cruz M, Lackman RD, Fox EJ (2010). Fibroma of tendon sheath in the knee a report of three cases and literature review. Knee..

[16] Yeoh HJ, Kim T-Y (2019). The feasibility of shear wave elastography for diagnosing superficial benign soft tissue masses. Ultrasonography..

[17] Sookur PA, Saifuddin A (2011). Indeterminate soft-tissue tumors of the hand and wrist a review based on a clinical series of 39 cases. Skeletal Radiologh.

[18] Lelong C, Delpierre I, Remmelink M, Verbeurgt C (2021). Fibroma of the tendon sheath of the neck. Eur Ann Otorhinolaryngol Head Neck Dis.

[19] Suzuki K, Yasuda T, Suzawa S, Watanabe K, Kanamori M, Kimura T (2017). Fibroma of tendon sheath around large joints: clinical characteristics and literature review. BMC Musculoskelet Disord.

[20] Hamdi MF, Touati B (2012). Giant cell tumour of the flexor tendon sheath of the hand analysis of 27 cases. Musculoskelet Surg.

[21] Aynaci O, Kerimoglu S, Ozturk C, Saracoglu M, Yildiz K (2009). Intraarticular fibroma of the tendon sheath arising from the infrapatellar fat pad in the knee joint. Arch Orthop Trauma Surg.

